# Estimation of cardiorespiratory fitness using heart rate and step count data

**DOI:** 10.1038/s41598-023-43024-x

**Published:** 2023-09-22

**Authors:** Alexander Neshitov, Konstantin Tyapochkin, Marina Kovaleva, Anna Dreneva, Ekaterina Surkova, Evgeniya Smorodnikova, Pavel Pravdin

**Affiliations:** Welltory Inc., 541 Jefferson, Suite 100, Redwood City, CA 94063 USA

**Keywords:** Engineering, Physiology

## Abstract

Predicting cardiorespiratory fitness levels can be useful for measuring progress in an exercise program as well as for stratifying cardiovascular risk in asymptomatic adults. This study proposes a model to predict fitness level in terms of maximal oxygen uptake using anthropometric, heart rate, and step count data. The model was trained on a diverse cohort of 3115 healthy subjects (1035 women and 2080 men) aged 42 ± 10.6 years and tested on a cohort of 779 healthy subjects (260 women and 519 men) aged 42 ± 10.18 years. The developed model is capable of making accurate and reliable predictions with the average test set error of 3.946 ml/kg/min. The maximal oxygen uptake labels were obtained using wearable devices (Apple Watch and Garmin) during recorded workout sessions. Additionally, the model was validated on a sample of 10 subjects with maximal oxygen uptake determined directly using a treadmill protocol in a laboratory setting and showed an error of 4.982 ml/kg/min. Unlike most other models, which use accelerometer readings as additional input data, the proposed model relies solely on heart rate and step counts—data readily available on the majority of fitness trackers. The proposed model provides a point estimation and a probabilistic prediction of cardiorespiratory fitness level, thus it can estimate the prediction’s uncertainty and construct confidence intervals.

## Introduction

Cardiorespiratory fitness (CRF) is a key indicator of both athletic performance and general health. CRF characterizes the functional ability of the lungs, cardiovascular system, and skeletal muscles to perform daily activities that require a sustained aerobic metabolism^1^, and is inversely correlated with several pathologies (e.g. atherosclerosis, heart failure)^2, 3^. A widely recognized measure of an individual’s CRF is the maximal oxygen uptake (VO2 max). It was introduced by Hill and Lupton in a 1923 paper^4^ as “the oxygen intake during an exercise intensity at which actual oxygen intake reaches a maximum beyond which no increase in effort can raise it”. The methods to estimate a person’s VO2 max level can be divided in two groups: direct and indirect methods^5^. Direct methods typically involve a graded exercise test conducted in a controlled laboratory setting with direct readings of oxygen consumption and include reaching a maximal level of a person’s physical activity. Most common direct methods are treadmill tests where the subject runs on a treadmill with an increasing gradient or speed, or bicycle ergometer tests where individual pedals on a stationary bike with the resistance or pedaling rate gradually increasing. During these tests the measurement of oxygen consumption increases with the intensity of exercise until it reaches a plateau or the subject cannot maintain the required intensity, indicating the VO2 max level. Validity of these methods was verified in several research studies^6–9^.

Indirect methods, such as Astrand treadmill test^10^ or Astrand-Ryhming cycle ergometer test^11^ do not require measurements of oxygen consumption during exercies. Instead, they use heart rate response to a standard workload and do not require reaching maximal intensity^12^. While direct methods for VO2 max estimation are more accurate, they are also costlier and require more time for setup and operation by trained technicians. On the other hand, indirect methods are more accessible and less time-consuming, but their accuracy can be lower as they use interpolation to estimate maximal oxygen uptake rather than directly measuring it^13^.

Wearable devices have made it possible to track heart rate and physical exertion not only in a laboratory setting, but also in everyday life. The use of wearable devices as physical activity and health tracking tools has increased over the last five years, both among professional and non-professional athletes^14^. A large number of wearable sensors and mobile applications analyze various physiological indicators, such as heart rate, step count, and distance traveled. Heart rate is frequently used to estimate cardiovascular strain during exercise^15^. A lower heart rate at a given workload is associated with higher cardiorespiratory fitness^16^. Previous research demonstrates the feasibility of using physiological data obtained from electronic devices to predict VO2 max. Various features were used: speed to heart rate ratio in^17^; contextualized heart rate in^18–20^; heart rate response features collected during controlled submaximal treadmill test in^21^; variability in the daily traveled distance in^22^. Several smartwatches, such as Apple Watch and Garmin, estimate VO2 max for every sufficiently long walking or running session using proprietary algorithms that analyze heart rate, accelerometer, and GPS data collected during the session. The accuracy of the estimates was validated in the manufacturer’s whitepapers^23, 24^.

The objective of this study is to develop a machine learning model capable of making reliable VO2 max predictions using heart rate and step count interval data collected from users in everyday life. These data sources have been chosen since they can be supplied by the majority of wearable devices and do not necessitate separate tracking of running or walking sessions. To do this, a dataset containing a history of heart rate measurements and step count intervals from a diverse cohort of wearable device users was collected. The dataset was split in train and test part to train and validate the model. Additionally, the model was validated on a small dataset of volunteers with VO2 max values measured directly in laboratory settings.

The paper is organized as follows. Section Data collection describes the data collection process. Section Features discusses the process of feature engineering. Section Model describes the model construction and tuning process. Section Results analyzes the model accuracy for probability and point prediction, provides the analysis of feature importance, and analyzes how each of the features contributes to the prediction using the Shapley values. Section Comparison with direct VO2 max observations contains a comparison of the model prediction results with VO2 max data determined in a laboratory setting. Section Discussion provides an overview of the obtained results, model limitations, and directions for future research.

## Materials and methods

### Study design

In this study, VO2 max is considered as the rate relative to body mass and expressed in ml/kg/min. Two datasets were collected:

- Wearable devices dataset: a dataset containing history of everyday step count data and heart rate data collected in background by smartwatch devices (Apple Watch and Garmin) from a diverse cohort of users, together with VO2 max estimates made by smartwatch algorithms during intentionally tracked workout sessions.
- Direct VO2 max dataset: a dataset containing history of everyday step count data and heart rate data collected in background by Apple Watch devices from ten volunteers, who participated in direct VO2 max estimation in a laboratory setting.

The wearable devices dataset was split into a train and test set. A machine learning model was trained on users’ history of heart rate and step count data in the train set, then its performance was tested by comparing its predicitons with labels on the test set. Its predictions were compared with VO2 max values on the direct VO2 max dataset.

### Data collection

Smartwatch data collection was conducted without the active participation of the research subjects. Upon downloading the Welltory app, users provide informed consent for their anonymized data to be used by the company for internal research purposes if such research can help provide users with better services or improve the app’s functionality. This policy is described in the company’s Terms of Use, which the app’s users actively consent to.

User data was gathered between January 2020 and October 2021 in a non-controlled setting. The data collected from wrist-worn smartwatches (Apple Watch and Garmin), synchronized with the Welltory app, includes gender, age, height, weight, heart rate measurements, and intervals with step-count data provided by each device’s internal algorithms. VO2 max values estimated by the devices’ internal algorithms during outdoor walking or running workout sessions were also gathered. Apple Watch and Garmin devices measure heart rate ranging from once per several minutes in the background to once per several seconds during a separately tracked workout session. In order to ensure the developed model remains independent from device-specific measurement frequency, the obtained heart rate stream is resampled to one measurement per minute (for the minutes containing measurements) during the preprocessing stage (see Section Preprocessing for more detail). VO2 max labels were obtained by gathering VO2 max estimates made by Apple Watch and Garmin devices during running and walking sessions, with the average taken for each study participant.

### Wearable devices dataset

Data collected from 3894 subjects was split into a train(80%) and test(20%) set. Table 1 provides descriptive statistics of the train and test datasets.

**Table 1:** Wearable devices dataset characteristics.

	Train set (N $=$ 3115)	Test set (N $=$ 779)
Gender distribution:		
Female	1035 (33%)	260 (33%)
Male	2080 (67%)	519 (67%)
Age distribution:		
$\le$ 20 years	13 (0.4%)	3 (0.4%)
20–30 years	351 (11.3%)	72 (9.2%)
30–40 years	1057 (33.9%)	247 (31.7%)
40–50 years	961 (30.9%)	269 (34.5%)
50–60 years	556 (17.8%)	148 (19.0%)
$> 60$ years	177 (5.7%)	40 (5.1%)
Body mass index categories:		
Underweight (BMI $< 18.5$)	55 (1.8%)	5 (0.6%)
Normal weight (18.5 $\le$ BMI $< 25.0$)	1258 (40.4%)	321 (41.2%)
Overweight (25.0 $\le$ BMI $< 30.0$)	1179 (37.8%)	287 (36.8%)
Obese (BMI $\ge$ 30.0)	623 (20.0%)	166 (21.3%)
Devices:		
Apple Watch	2912 (93.5%)	726 (93.2%)
Garmin	203 (6.5%)	53 (6.8%)
Number of observed days (mean ± SD)	287±149	288±149
Reference VO2 max (ml/kg/min, mean ± SD)	36.16±6.66	36.18±6.76
Number of VO2 max measurements (mean ± SD)	8.69±5.43	8.96±5.53

In the collected dataset the VO2 max labels are derived from estimates made by wearable devices during intentionally tracked workout sessions. Since these labels may deviate from actual VO2 max values, a smaller dataset containing VO2 max values obtained using direct methods in laboratory conditions was collected:

### Direct VO2 max dataset

Ten healthy Apple Watch users volunteered for laboratory tests to determine their maximal oxygen uptake. Everyday heart rate and step count data, gathered from these volunteers’ Apple Watches in the month preceding the testing, served as the input for the developed model. The model’s predictions were then compared to maximal oxygen uptake obtained during the laboratory testing.

Each subject provided informed consent. The exercises were performed under a supervision of a trained exercise physiologist. The study was conducted in accordance with the principles of the Declaration of Helsinki and approved by the ethics committee of the South Ural State University. Characteristics of the participants are given in Table 2.

**Table 2:** Direct VO2 max validation study participants.

	Women (N $=$ 6)	Men (N $=$ 4)	All (N $=$ 10)
Age, years (mean ± SD)	32.3 ± 4.5	35.6 ± 5.5	33.6 ± 5.2
Height, cm (mean ± SD)	165.7 ± 10.1	178.8 ± 5.1	170.9 ± 10.6
Weight, kg (mean ± SD)	61.3 ± 6.6	77.8 ± 13.8	67.9 ± 12.9
BMI, kg/$\text {m}^2$ (mean ± SD)	22.4 ± 2.7	24.2 ± 3.3	23.2 ± 3.1
VO2 max, ml/kg/min (mean ± SD)	35.1 ± 4.5	41.4 ± 3.9	37.6 ± 5.2

#### Validation dataset collection protocol

Each participant was performing a treadmill exercise while wearing a MetaMax 3B (R2) device measuring the oxygen consumption together with a chest strap measuring the heart rate during the exercise. The accuracy of the MetaMax 3B device was validated in an independent study^25^. Oxygen consumption (VO2) was measured every 2 seconds.

Each exercise session initiated with a starting treadmill speed of 5 km/h. Following a 3-minute warm-up, the treadmill speed was incrementally increased by 0.1 km/h every 6 seconds until the participant’s oxygen consumption plateaued or the participant could no longer sustain the required treadmill speed. The VO2 peak value was obtained as the highest VO2 value averaged over 40-second intervals. The selection of a 40-second window is based on its use in literature^6^, and its effectiveness in reducing noise in VO2 measurements.

Out of 10 volunteers, 8 exhibited a plateau in their VO2 consumption. This plateau is defined by a VO2 increase of less than 50% of the anticipated rise for the corresponding increase in work rate^26^. The 2 volunteers who did not show this plateau pattern achieved a respiratory exchange ratio greater than 1.1, and peak heart rate above 95% of the age predicted maximums. As the result, their VO2 peak measurements were considered accurate estimates of VO2 max according to commonly used criteria^27^. An example of the recorded data is shown in Fig. S1.

### Features

History of heart rate and step count data for each user was transformed to a feature vector using the following procedure.

#### Preprocessing

The heart rate stream is given as a sequence of timestamps and heart rate values. To avoid the influence of device-dependent heart rate reading frequency, the heart rate stream is averaged over consecutive 1-min intervals. This gives a sequence $(t_i, hr_i)$ where $t_i$ are entire minute timestamps and $hr_i$ is the average of heart rate readings during the minute starting on $t_i$. The step count data is given as a sequence of intervals $(start_j, end_j, stepcount_j)$ indicating the number of steps made in the interval $(start_j, end_j)$. The average cadence $c_i$ (expressed in steps per minute) is computed at moments $t_i$ as the number of steps made during the 1-min interval ending at $t_i$. Finally, this gives a sequence $t_i, hr_i, c_i$ of timestamps, heart rate, and cadence data. These sequences are then used to derive the input features for the model.

#### Cadence to heart rate ratio

Previous research highlighted the importance of speed to heart rate ratio as a feature for VO2 max prediction^17^. Without explicit speed and distance data available, the cadence to heart rate ratio serves as a substitute. For a specific heart rate and cadence sequence, minutes containing activity are considered, i.e., having a cadence $c_i > 60$. Let *chr* denote the distribution of the ratio of cadence to heart rate during activity minutes. Let $chr_{25}, chr_{50}, chr_{75}$ denote three quartiles of the distribution *chr*. Consider the vector of quartiles

1 $$\begin{aligned} x_{chr} = (chr_{25}, chr_{50}, chr_{75}) \end{aligned}$$

The vector $x_{chr}$ is one of the input features.

#### Daily MET-minutes

Another feature estimates an individual’s overall physical activity. This involves the use of the metabolic equivalent of task (MET), the ratio of the oxygen consumption at a specific moment to the oxygen consumption at rest, generally accepted to be equal to 3.5 ml/kg/min^28^. An estimate of MET is derived based on heart rate using the method developed in^29^:

2 $$\begin{aligned} \text {MET} = 6 \cdot \frac{hr}{hr_{rest}} - 5 \end{aligned}$$

Here, *hr* represents the heart rate at a certain moment, and $hr_{rest}$ is the individual’s heart rate at rest on a given day, computed as the $10\%$-percentile of the heart rate recorded between noon and 9 pm of that day. For every day in an individual’s measurement history, the MET values computed for all minutes during that day are added up. Minutes not covered by heart rate readings are assumed to have a MET value of one. The computed MET-minutes are then divided by 1440, corresponding to MET-minutes of a day without any activity. Thus, a single number *met* is obtained for every observed day. Three quartiles of the distribution *met* for all observed days are computed and these quartiles are denoted by $met_{25}, met_{50}, met_{75}$. The vector consisting of these quartiles is denoted by $x_{met}$:

3 $$\begin{aligned} x_{met} = (met_{25}, met_{50}, met_{75}). \end{aligned}$$

$x_{met}$ is then utilized as a feature describing the usual level of physical activity of the individual.

#### Heart rate response to cadence increase

The next feature highlights how an individual’s heart rate reacts to an increase in cadence. For the cadence and heart rate sequence $c_i, hr_i$, active moments with $hr_i$ above 75 bpm and $c_i$ over 60 steps per minute are considered. The threshold of 75 bpm for heart rate is used, since it corresponds to 2.5 MET using Eq. (2) for resting heart rate of 60 bpm, a generally accepted lower bound for resting heart rate in adults^30^. The study^31^ models the relationship between cadence and MET using a continuous function consisting of two linear segments. Utilizing the nearly linear relationship between oxygen consumption and heart rate^29^, the relationship between heart rate and cadence is modeled using a continuous two-segment piecewise linear function as well. Piecewise linear functions with the node at the cadence of 100 steps per minute are used. The node of two-segment piecewise linear function is fixed at cadence level of 100 steps per minute as it corresponds to MET=3 and is a breakpoint in MET-cadence regression in^31^.

A continuous two-segment piecewise linear function is determined by three parameters: the intercept *w*0, the slope of the first linear segment *w*1, and the slope of the second segment *w*2. For robust estimates, quantile regression is performed by fitting the coefficients *w*0, *w*1, *w*2 to minimize the pinball loss (refer to Section Quantile Regression for more detail) for various quantile levels (see Fig. S2 for example of fitted piecewise quantile functions).

To illustrate how the position and shape of the fitted quantile regression aids in predicting VO2 max, Fig. S3 contains the median fitted piecewise quantile curves to (cadence, heart rate) distributions for participants with low (below the 1/3 quantile in the training set, red area), medium (between 1/3 and 2/3 quantiles in the training set, yellow area) and high (above the 2/3 quantile in the training set, green area) values of VO2 max. One can see that on average people with higher values of VO2 max have lower heart rate for the same cadence. Also, one can see that the slope of the heartrate-over-cadence regression line is lower for people with high VO2 max (green group) vs people with low VO2 max (red group). This is especially visible in the region with cadence between 60 and 100 steps per minute. In the higher cadence range, while one can still observe the lower level of heart rate for people with higher VO2 max, the distinction in the growth rate becomes less visible.

Quantile levels 0.1, 0.2, 0.5, 0.8, 0.9 were chosen for the heart rate on cadence regression. For each quantile level, three coefficients *w*0, *w*1, *w*2 are obtained, culminating in a total of 15 parameters from $w0_{10}, w1_{10}, w2_{10}$ (coefficients of quantile regression for $q=0.1$) to $w0_{90}, w1_{90}, w2_{90}$ (coefficients of quantile regression for $q=0.9$). These coefficients collectively form a vector that characterizes the heart rate response to an increase in cadence:

4 $$\begin{aligned} x_{resp} = (w0_{10}, w1_{10}, w2_{10},\ldots w0_{90}, w1_{90}, w2_{90}) \end{aligned}$$

#### Anthropometric features

In addition to the features derived from the heart rate and cadence sequences, gender, age, and the body mass index:

5 $$\begin{aligned} \text {bmi} = \frac{\text {weight in kg}}{(\text {height in m})^2} \end{aligned}$$

are joined into the vector of anthropometric features:

6 $$\begin{aligned} x_{ant} = (\text {age}, \text {gender}, \text {bmi}) \end{aligned}$$

Finally, the feature vectors described above are concatenated into one 24-dimensional vector *x* that serves as an input of the developed model:

7 $$\begin{aligned} x = (x_{ant}, x_{chr}, x_{met}, x_{resp}) \end{aligned}$$

### Model

#### Quantile Regression

If *Y* is a random variable with cumulative distribution function $F_Y$, and $0< q < 1$, let $Q_Y(q)=F_Y^{-1}(q)$ denote the *q*-th quantile of *Y*. The function^32^

8 $$\begin{aligned} L_q(y, {\hat{y}}) = q\max (y - {\hat{y}}, 0) + (1-q)\max ({\hat{y}} - y, 0) \end{aligned}$$

is called pinball loss. If *Y* is a random variable, its *q*-th quantile minimizes the expected pinball loss $E(L_q(Y,-))$^33^:

$$\begin{aligned}Q_Y(q) = \mathop {{\text {argmin}}}_{z}E(L_q(Y, z))\end{aligned}$$

Thus if *X* is a random vector and *Y* is a random variable, the conditional quantiles $Q_{Y\mid X=x}(q)$ minimize the expected pinball loss $E(L_q(Y, f(X)))$ among all functions *f* mapping the range of *X* to the range of *Y*. Therefore, for a train set $(x_i, y_i)_{i=1}^n$ quantile regression aims to find a function *f*(*x*) that minimizes the total loss

$$\begin{aligned}\sum _{i=1}^n L_q(y_i, f(x_i)).\end{aligned}$$

Quantile regression was introduced in^32^ for linear functions *f*. Later quantile regression was studied for other models, such as gradient boosting machines^34^, random forests^35, 36^, and neural networks^37^.

#### VO2 max probability prediction

Let (*X*, *Y*) denote the joint distribution of feature vectors and VO2 max labels. The developed model provides estimates ${\hat{Q}}(x,q)$ of the conditional quantiles $Q_{Y|X=x}(q)$ for $0< q < 1$ using the following construction. For every $q=0.05, 0.1,\ldots , 0.95$ a gradient boosting model $f_q(x)$ that minimizes the pinball loss $\sum _{i=1}^n L_q(y_i, f_q(x_i))$ is fitted on a training set $(x_i, y_i)_{i=1}^n.$

Since the models are fitted independently, the sequence $f_{q}(x)$ does not necessarily guarantee to be non-increasing with respect to *q*. To ensure monotonicity of estimates $f_q(x)$ and acquire a more robust estimate, the rearrangement operation introduced in^38^ is applied. Specifically, for every *x*, a function $f_x:[0, 1]\rightarrow {\mathbb {R}}$ is constructed by interpolating values $f_q(x)$ for $q=0.05, \ldots , 0.95$. Here, $U~\sim U(0,1)$ denotes the random variable uniformly distributed on the interval [0, 1]. Denote by $f^*_x$ the quantile function of the random variable *f*(*U*). The function $f^*_x$ is called the rearrangement of the function $f_x$. Finally, the model prediction of the *q*-th conditional quantile is defined as the value of rearrangement function $f^*_x$ at level *q*:

9 $$\begin{aligned} {\hat{Q}}(x,q) = f^*_x(q) \end{aligned}$$

By^38^, Proposition 1 the estimates ${\hat{Q}}(x,q)$ are closer to the true conditional quantiles $Q_{Y|X=x}(q)$ than the initial estimates $f_q(x)$, and ${\hat{Q}}(x, q) \leqslant {\hat{Q}}(x,q')$ when $q\leqslant q'$

#### Model training and hyperparameter tuning

To estimate the probability prediction model, the continuous ranked probability score (CRPS)^39^ is used. CRPS is defined for a probabilistic prediction with cumulative distribution function *F* and observed value *y* as

10 $$\begin{aligned} CRPS(F,y) = -\int _{-\infty }^{\infty } (F(z) - 1\{z\geqslant y\})^2 dz \end{aligned}$$

To use this metric with predicted quantiles, for every feature-label pair (*x*, *y*) the cumulative distribution function *F* is recovered as the inverse of the estimated quantile function ${\hat{Q}}(x, -)$. or each potential set of hyperparameters, a 5-fold cross-validation on the training set is performed to estimate the average CRPS score. Hyperparameters are then tuned to maximize the cross-validated CRPS score using the tree-structured Parzen Estimator algorithm implemented^40^. The gradient boosting models were trained using LightGBM implementation^41^. The tuning gives the following optimal choice of the gradient boosting hyperparameters given in Table 3:

**Table 3:** Optimal parameters of gradient boosting models.

Maximal tree depth	4
Number of estimators	313
Bagging fraction	0.75
Bagging frequency	13
Learning rate	0.1
Minimal number of samples in leaf	22
$L_1$ regularization coefficient	0.142

### Metrics

Estimating the accuracy of probabilistic predictions requires evaluation of calibration and sharpness^42^. Calibration demonstrates the differences between the quantiles predicted by the model and the true quantiles of the conditional distribution $F_{y|x}$, while sharpness estimates the spread of the predicted distribution.

#### Calibration

Given the lack of information about the distribution of the label for a specific feature (since typically there is only one example with the given value of the feature vector *x*), the calibration is estimated using the expected calibration error^43^, defined as follows.

For a feature-label set $(x_1, y_1), \ldots (x_n, y_n)$ and $0< q < 1$ consider the probability of observing a sample below the predicted quantiles:

11 $$\begin{aligned} p^{obs}(q) = \frac{1}{n}\sum _{i=1}^n 1\{y_i\leqslant {\hat{Q}}(x_i, q)\} \end{aligned}$$

The *expected calibration error* is defined as the average difference between the observed probabilities and the quantile level:

12 $$\begin{aligned} ECE({\hat{Q}}) = \int _0^1|p^{obs}(q)-q|dq \end{aligned}$$

#### Sharpness

Note that the expected calibration error does not tell how informative the model is, since the constant model predicting marginal quantiles of the label *y* regardless of the feature *x* will have the expected calibration error equal to zero. Therefore in addition to calibration, it is necessary to estimate the sharpness of the model’s predictions, that indicates how informative the predicted distributions are.

For the model ${\hat{Q}}$ and a set of feature vectors $x_1,\ldots , x_n$ the *sharpness* of the model prediction is estimated by the average interquartile range (IQR) of the predicted distributions:

13 $$\begin{aligned} IQR({\hat{Q}}) = \frac{1}{n}\sum _{i=1}^n{\hat{Q}}(x_i, 0.75) - {\hat{Q}}(x_i, 0.25) \end{aligned}$$

### Shapley values

To estimate the impact of different features on the predictions, the Shapley values^44^ are used. For a component of a feature vector, its Shapley value shows what contribution does this component bring to the prediction. In informal terms, a Shapley value of a component shows the difference between the prediction when the component value is known and the prediction when the component value is unknown. In particular, if a change in a component does not affect the prediction, its Shapley value is zero, and the components that are more relevant for prediction have larger Shapley values than less relevant components.

To estimate how different features impact the model prediction, the Shapley values (implemented in^45^ for gradient boosting models) are computed for the median prediction model $f=f_{0.5}$. Let us briefly recall the definition of the Shapley values. Suppose *x* is a feature vector. Enumerate its components as $x=(x^{(1)},\ldots , x^{(m)})$. Let ${\underline{m}}$ denote the set $\{1,2,\ldots , m\}$ of all input features. If $S\subseteq {\underline{m}}$ is a subset of features, let $x^S$ denote the subvector of *x* consisting of components in *S*. If *x* and *y* are two feature vectors, then $(x^S, y^{{\underline{m}}\setminus S})$ denotes the feature vector *z* defined as

$$\begin{aligned}z^{(i)} = {\left\{ \begin{array}{ll} x^{(i)} \text { if } i \text { is in } S \\ y^{(i)}\text { if } i \text { is not in } S \end{array}\right. }\end{aligned}$$

If $x_1, \ldots x_n$ denote the feature vectors in the training set. For any feature vector *x* denote by $f_S(x)$ the average value of *f* on the training set where features in *S* were set to be equal to $x^S:$

14 $$\begin{aligned} f_S(x) = \frac{1}{n}\sum _{i=1}^{n}f(x_i^{{\underline{m}}\setminus S}, x^S) \end{aligned}$$

Thus $f_{{\underline{m}}}(x)$ coincides with *f*(*x*) and $f_{\emptyset }(x)$ is the average value of *f* on the training set.

Then the Shapley value of the *i*-th feature in the input vector *x* is defined as

15 $$\begin{aligned} \phi _i(x) = \sum _{S\subseteq {\underline{n}}\setminus \{i\}}\frac{|S|!(n-|S|-1)!}{n!}(f_{S\cup \{i\}}(x) - f_S(x)) \end{aligned}$$

It represents an impact of the *i*-th feature on the model at the point *x*. The model value *f*(*x*) decomposes as the average value on the training set and the sum of Shapley values at *x*

16 $$\begin{aligned} f(x) = \frac{1}{n}\sum _{i=1}^nf(x_i)+\sum _{i=1}^m \phi _i(x) \end{aligned}$$

## Results

### Calibration and sharpness

Table 4 shows the calibration and sharpness of the model on the test set. For comparison purposes, the metrics for models trained using various subsets of features are reported.

**Table 4:** Calibration and sharpness of the probability prediction model on the test set.

Features used in model ${\hat{Q}}$	$ECE({\hat{Q}})$	$IQR({\hat{Q}})$
None	0.003	8.874
$x_{ant}$	0.026	6.279
$x_{ant}, x_{chr}$	0.039	4.665
$x_{ant}, x_{chr}, x_{resp}$	0.029	4.362
$x_{ant}, x_{chr}, x_{resp}, x_{met}$	0.032	3.948

Calibration and sharpness on test set for models trained with various subsets of features.

To simplify the comparison with models giving point estimates of VO2 max, the Table 5 contains the error metrics of the median prediction ${\hat{Q}}(x, 0.5)$ made by the developed model trained using various subsets of features:

**Table 5:** Error of median prediction $y - {\hat{Q}}(x, 0.5)$ (mean ± SD).

Features used	Train set	Test set
None	− 0.137 ± 6.661	0.032 ± 6.760
$x_{ant}$	0.084 ± 4.880	0.424 ± 5.392
$x_{ant}, x_{chr}$	0.158 ± 3.878	0.261 ± 4.487
$x_{ant}, x_{chr}, x_{resp}$	0.145 ± 3.324	0.083 ± 4.143
$x_{ant}, x_{chr}, x_{resp}, x_{met}$	− 0.082 ± 3.052	0.013 ± 3.946

Error of median prediction of the model trained using different subsets of features.

### Feature impact

Recall that feature vectors consist of the anthropometric features, cadence to heart rate ratio features, MET-features and response features:

$$\begin{aligned}x=(x_{ant}, x_{chr}, x_{met}, x_{resp})\end{aligned}$$

For every feature group $x_{ant}, x_{chr}, x_{met}, x_{resp}$ define its Shapley value at *x* the sum of individual Shapley values of the features in that group. Thus the mean absolute Shapley value of a feature group is an indicator of its importance for the model predictions. Table 6 shows the obtained feature group importance.

**Table 6:** Feature group importance.

Feature group	Mean absolute Shapley value
$x_{ant}$	2.766
$x_{chr}$	1.545
$x_{resp}$	1.187
$x_{met}$	0.755

The Shapley values for each individual are depicted in Fig. S4. The figure reveals the following impacts of main features: older age and higher BMI correlate with lower cardiorespiratory fitness. Larger cadence to heart ratio values $chr_{75}$ and greater levels of physical activity as shown by $met_{75}$ correlate with higher cardiorespiratory fitness. Regarding the features expressing the heart rate response to the increase in cadence, it is observed that elevated values of intercept ($w0_{50}$) and the first segment slope $w1_{50}$ also correlate with lower cardiorespiratory fitness. Specifically, the plot of Shapley values for $w0_{50}$ demonstrates that values of $w0_{50}$ below the *y*-axis intercept point of approximately 95 positively impact the prediction, while values of $w0_{50}$ above that point negatively impact the prediction. This suggests the learned association between elevated levels of heart rate during walking (corresponding to higher $w0_{50}$) and lower levels of VO2 max, a dependence depicted in Fig. S3. A similar conclusion can be drawn about the feature $w1_{50}$. At the same time, the individual impact of the second segment slope $w2_{50}$ is less pronounced as the feature $w2_{50}$ operates in conjunction with features *w*0 and *w*1. The influences of features on the sharpness of the model’s predictions are presented in Fig. S5. It illustrates that low values of the second segment slope $w2_{50}$ and the cadence to heart rate ratio feature $chr_{75}$ contribute to larger uncertainty in the model’s predictions. A potential explanation for this behavior is that samples with low $w2_{50}$ and $chr_{75}$ derive from data with few recorded moments of large cadence. In such situations, the model has less information on the individual’s response to intense physical activity, leading to less certainty in its predictions.

#### Dependence of the model’s prediction sharpness on the amount of available data

It is expected that the model’s prediction sharpness should decrease when more input data becomes available. To validate this conjecture, the following steps were performed for every user in the test set:

- For every month containing a reference smartwatch VO2 max label consider history of heart rate measurements and step count data in the intervals of 1 week, 2 weeks, 1 month, 4 months and 8 months preceding the time when the label was obtained.
- For each interval compute the number of minutes of activity (i.e. minutes with cadence above 60 steps per minute) and then compute the model predictions based on the data from the interval. Then calculate the model’s prediction sharpness, defined as the predicted interquartile range, i.e. the difference between the upper and lower conditional quartiles predicted by the model.

Table 7 and Fig. S6 show how the model’s predicted sharpness depends on the amount of available active minutes in the user’s history. They show how the model’s prediction uncertainty gradually decreases with the increase of the available data.

**Table 7:** Sharpness of the model’s predictions depending on the amount of available data.

Active minutes in user data	IQR(${\hat{Q}}$)
Less than 10	7.39
10 to 30	6.25
30 to 60	5.63
60 to 100	5.22
100 to 200	4.92
200 to 500	4.54
500 to 1000	4.25
more than 1000	3.97

#### Model accuracy comparison for Apple Watch and Garmin devices

To compare how the developed model perfoms on data obtained from different devices, calibration and sharpness metrics for test samples were computed separately for data collected using Apple Watch devices and Garmin devices. The Table 8 shows expected calibration error and interquartile range for the test set samples collected from Apple Watch and Garmin devices.

**Table 8:** Calibration and sharpness on the test set.

Source	$ECE({\hat{Q}})$	$IQR({\hat{Q}})$
Apple Watch (N=726)	0.037930	3.904
Garmin (N=53)	0.037934	3.994

Mean expected calibration error and interquartile range computed separately for test samples collected using Apple Watch devices and Garmin devices.

Thus the model shows similar calibration and sharpness metrics on data samples collected using different devices.

## Comparison with direct VO2 max observations

### Model prediction

For each participant, Table 9 contains maximal oxygen uptake values determined using the treadmill test, and the quartiles predicted by the model using their heart rate and step count history.

**Table 9:** Comparison with VO2 max determined in laboratory tests.

Subject	Gender	Age	bmi	VO2 max	$\text {q}_{25}$	$\text {q}_{50}$	$\text {q}_{75}$
1	f	27	27.30	35.17	32.65	36.39	38.42
2	f	28	24.22	34.95	37.53	38.66	39.56
3	m	30	24.25	35.36	37.02	40.65	43.46
4	m	30	22.38	44.69	35.76	37.13	42.09
5	f	31	22.89	39.12	31.97	34.67	35.88
6	f	32	21.12	40.11	35.84	37.94	41.65
7	f	41	18.63	26.20	33.89	34.88	36.13
8	m	36	29.59	44.92	36.68	38.46	40.40
9	f	32	20.28	35.32	34.43	35.54	37.91
10	m	44	20.69	40.48	35.40	36.78	40.41

VO2 max values found in laboratory treadmill tests, and three quartiles predicted by the model.

The calibration and sharpness of the model prediction as well as error of the median prediction on the laboratory dataset are given in Table 10.

**Table 10:** Metrics for model prediction on the laboratory dataset participants.

Features used	$ECE({\hat{Q}})$	$IQR({\hat{Q}})$	Median error (mean ± SD)
None	0.100	8.201	1.191 ± 5.231
$x_{ant}$	0.058	6.590	0.622 ± 5.551
$x_{ant}$, $x_{chr}$	0.084	4.940	0.324 ± 5.148
$x_{ant}$, $x_{chr}$, $x_{resp}$	0.111	4.779	0.897 ± 5.039
$x_{ant}$, $x_{chr}$, $x_{resp}$, $x_{met}$	0.084	4.705	0.349 ± 4.982

## Discussion

The development of a simple, inexpensive, and effective method to assess CRF has been recognized by the American College of Sports Medicine as a matter of paramount importance^46^. Such a method will make it possible to identify and mitigate the negative health effects of low CRF caused by a lack of physical activity. The maximal oxygen uptake (VO2 max) is a common measure of CRF. It is an important indicator of the body’s ability to tolerate aerobic exercise and a good predictor of endurance^47^. Moreover, VO2 max allows the assessment of training progress and cardiovascular risk stratification in asymptomatic adults, as low VO2 max increases the risk of all-cause mortality, including cardiovascular events^48^. The limitations of previously used laboratory maximal and submaximal exercise protocols for VO2 max assessment include their high cost, as well as the impossibility of large-sample testing and batch analysis. Besides, such exercise tests increase the risk of adverse events in asymptomatic individuals with unknown underlying cardiovascular and respiratory conditions^49^. Wearable devices allow us to investigate the relationship between physical activity, heart rate and VO2 max in a free-living environment. Furthermore, advances in signal processing and machine learning provide new opportunities for accurate VO2 max prediction based on physiological data acquired in free-living. In the present paper, a model for predicting VO2 max based on heart rate and step count was developed and tested. Previously, Altini et al. demonstrated the feasibility of using physiological data obtained from electronic devices to predict VO2 max during running^17^, walking^19^ and daily activities^20^. Algorithms developed in these works rely on accelerometer data used for heart rate contextualization. Current algorithms used in smartwatches^23, 24^ rely on accelerometer and distance measurements during separately tracked outdoor walking and running sessions.

Existing publicly available algorithms^17, 18, 20–22^ utilize linear regression and were validated using laboratory protocols on 48, 46, 32, 41, and 50 participants, respectively. Having a larger cohort of 3894 participants allows for the training of a more expressive model than linear regression. Moreover, this model doesn’t just offer a point prediction of VO2 max as in the previously published algorithms but also provides an estimate of the VO2 max probability distribution. This feature enables the estimation of prediction uncertainty for every participant and the construction of confidence intervals for VO2 max.

The goal of this study is to investigate whether it’s possible to use limited data - specifically, heart rate measurements and step-count intervals gathered in free-living conditions - to achieve comparable accuracy in estimating VO2 max. Given that data from uncontrolled environments can vary in quality and completeness, the developed model doesn’t merely predict the VO2 max level but also calculates the uncertainty of this prediction, allowing for the construction of confidence intervals. This is achieved by using quantile regression to predict the conditional distribution of VO2 max. Quantile regression was selected for its computational efficiency and the interpretability of its results, as the predicted conditional quantiles can be directly used to build confidence intervals for the prediction. Moreover, quantile regression comes with an established methodology for its evaluation in terms of calibration and sharpness metrics. It’s important to note that there are alternative approaches to quantify prediction uncertainty. For instance, bootstrapping of point prediction models is another method (see e.g.^50, 51^). It would be interesting to inverstigate whether bootstrapping-based uncertainty estimation methods can be effective for VO2 max prediction task.

Gradient boosting trees are chosen as the backbone of the developed model for its computation efficiency, robustness, and interpretability (e.g. a well-developed implementation of Shapley values for tree-based models) and its ability to process features of various nature, such as anthropometric data and abstract features characterizing heart rate response to cadence increase. Another option could be artificial neural networks. They were used by a number of methods developed in literature (see^52^) for analysis of the laboratory collected data. Potentially, they are able to learn relevant features from raw data. However, they could be more susceptible to noise and could require more data for training to prevent overfitting. It is interesting to see if they can be used to get a better-performing model on free-living data.

The model was trained on a dataset with labels given by wearable devices’ estimates of VO2 max. The accuracy of these estimates was assessed by device manufacturers and was reported as 4.7 ml/kg/min for Apple Watch^23^, Table 2 and 3.5 ml/kg/min for Garmin^24^. The developed model shows agreement with those labels with error S.D. of 3.9 ml/kg/min, on the test part of the dataset, see Table 5). To compare the model predictions with actual VO2 max data, a sample of VO2 max data determined using a treadmill protocol in a laboratory setting was collected. Additionally, the history of everyday heart rate and step count was collected for participants of the laboratory tests. This allowed to compare the model predictions based on the history of the participants’ data with their actual VO2 max values. On this sample, the model showed an error with mean 0.35 and S.D. of 4.98 ml/kg/min (Table 10).

The developed model has the expected calibration error of 0.032 and sharpness of 3.948 ml/kg/min (see Table 4) on the test part of the wearable devices dataset, and expected calibration error of 0.084 and sharpness of 4.705 ml/kg/min (see Table 10) on the direct VO2 max dataset. It is shown that the model gives more precise predictions to users with a longer history of recorded physical activity and less certain predictions to users with shorter history (see Table 7). Specifically, users require over 100 active minutes in their history to achieve predictions with an expected interquartile range under 5 ml/kg/min, 200 min for expected interquartile range of 4.5 ml/kg/min, and over 1000 active minutes for predictions with an expected range under 4 ml/kg/min.

The developed model is independent from specifics of the data collection device, as it performs similarly on samples collected using different devices (Table 8).

The ACSM cardiorespiratory fitness classification^53^, Table 4.7, uses percentiles of VO2 max level from the Fitness Registry and the Importance of Exercise National Database^54^ to classify the cardiorespiratory fitness level into six categories: very poor (below the database 20% percentile), poor(20%-40%), fair(40%-60%), good(60%-80%), excellent(80%-95%), and superior (above the 95% percentile). The average width of each group is 5.4 ml/kg/min for men and 4.1 ml/kg/min for women, with broader categories for the younger population and narrower categories for the older population. Thus the model sharpness is comparable with the average width of a cardiorespiratory fitness category.

Analysis of features impact shows that anthropometric characteristics (gender, age, body mass index) is the most influential feature, followed by cadence to heart rate ratio feature, heart rate response feature and MET feature (see Table 6). Impact of features on individual prediction is shown in Fig. S4. It shows negative impact of age and bmi on predicted VO2 max values, and positive impact of the physical activity level expressed as MET feature. A more detailed discussion of feature impact is given in Section Feature impact. Large impact of anthropometric features might be a consequence of the fact that the model was trained on the general population, and its accuracy might be lower for more diverse populations with higher VO2 max variance.

The metrics reported for the large dataset with VO2 max values given by wearable device estimates measures the model agreement with those estimates that can deviate from the actual VO2 max data. Testing on a small sample of real VO2 max data shows a comparable accuracy, however validation on a larger, more diverse dataset with VO2 max data would give a better understanding of the model accuracy.

### Limitations

Labels in the dataset used for training have the standard deviation (6.3 ml/kg/min for men and 5.6 ml/kg/min for women), which is considerably smaller than the standard deviation in the commonly used reference database of^54^ (11.1 and 9.1 ml/kg/min respectively). Therefore it is expected that the trained model tends to make predictions closer to the average, and its uncertainty might be higher in more specific populations like athletes. The model was trained and validated on a healthy population, therefore it is expected that for cardiac patients or in other situations when the produced heart rate and step count relationship features are not meaningful, the model will show large uncertainty like it does in the case of missing measurement history (see Table 7).

The features used in the model are derived from the joint distribution of cadence and heart rate during the moments of physical activity. Therefore its predictions might be non-informative for user with a short recorded history of everyday activity: as mentioned in the Discussion section, the users need to have at least 200 active minutes in their history to obtain predictions with interquartile range of 4.5 ml/kg/min.

While the model’s prediction average interquartile range comprises approximately 10% of the average VO2 max value for younger population. However, for the older population, this range increases to 20% of their average VO2 max. As a result, the model’s predictions can become less informative for older individuals. When the model’s predicted median VO2 max is used to categorize cardiorespiratory fitness based on ACSM guidelines^53^, it should be noted that the median’s error standard deviation, estimated as 4.98 ml/kg/min on direct VO2 max dataset translates to a range of 10 ml/kg/min (3 MET). This can introduce uncertainty in fitness category assignment. Consequently, if such categorization directs exercise prescriptions, these deviations can result in unsuitable recommendations regarding exercise intensity, type, and volume.

The developed model was validated on two datasets - the test part of the wearable devices dataset and the direct VO2 max dataset. While the large size of the wearable devices dataset allows a confident estimate of the model’s calibration and sharpness against the labels derived from wearable’s estimates of VO2 max, the limited size (N=10) of the direct VO2 max dataset makes it difficult to confidently estimate the model performance against the laboratory derived VO2 max estimates. The direct VO2 max dataset has further limitations. It only contains data from Apple Watch users and its gender ratio is 60% female and 40% male, whereas the wearable devices dataset has a 33% female and 67% male ratio.

### Directions for future research

An important feature in the developed model describes the heart rate response to cadence increase using the coefficients of quantile regression of heart rate on cadence. It is important to understand whether there are other, more efficient and robust methods to estimate the shape of the joint distribution of cadence and heart rate that provides more efficient features for VO2 max prediction, as these features may allow researchers to obtain informative VO2 max estimates with less amount of recorded history of physical activity.

## Conclusion

This article describes a model that estimates the user’s cardiorespiratory fitness level based on gender, age, body mass index, and a history of heart rate and step count data collected in free-living using wearable devices. The model estimates the probability distribution of the VO2 max level by predicting the conditional quantiles. The model’s accuracy is similar to the accuracy of estimates made by Apple Watch and Garmin wearable devices during separately tracked outdoor walking and running sessions using GPS and distance data. Probabilistic prediction of the model allows one to estimate the uncertainty of the prediction, and it is shown that the model’s uncertainty decreases when a longer input data history becomes available.

### Supplementary Information

Supplementary Figure S1.

Supplementary Figure S2.

Supplementary Figure S3.

Supplementary Figure S4.

Supplementary Figure S5.

Supplementary Figure S6.

## Data Availability

The data that support the findings of this study are available from Welltory Inc. but restrictions apply to the availability of these data, which were used under license for the current study, and so are not publicly available. Data are however available from the corresponding author (A.N.) upon reasonable request and with permission of Welltory Inc.

## Abbreviations

CRF: Cardiorespiratory fitness
MET: Metabolic equivalent of task
VO2 max: Maximal oxygen uptake
BMI: Body mass index
CRPS: Continuous ranked probability score
ECE: Expected calibration error
SD: Standard deviation
