# Supplementary figures and images for: Estimation of cardiorespiratory fitness using heart rate and step count data
Source: Sci Rep. 2023 Sep 22;13:15808. doi: 10.1038/s41598-023-43024-x (PMC10517160; doi:10.1038/s41598-023-43024-x)

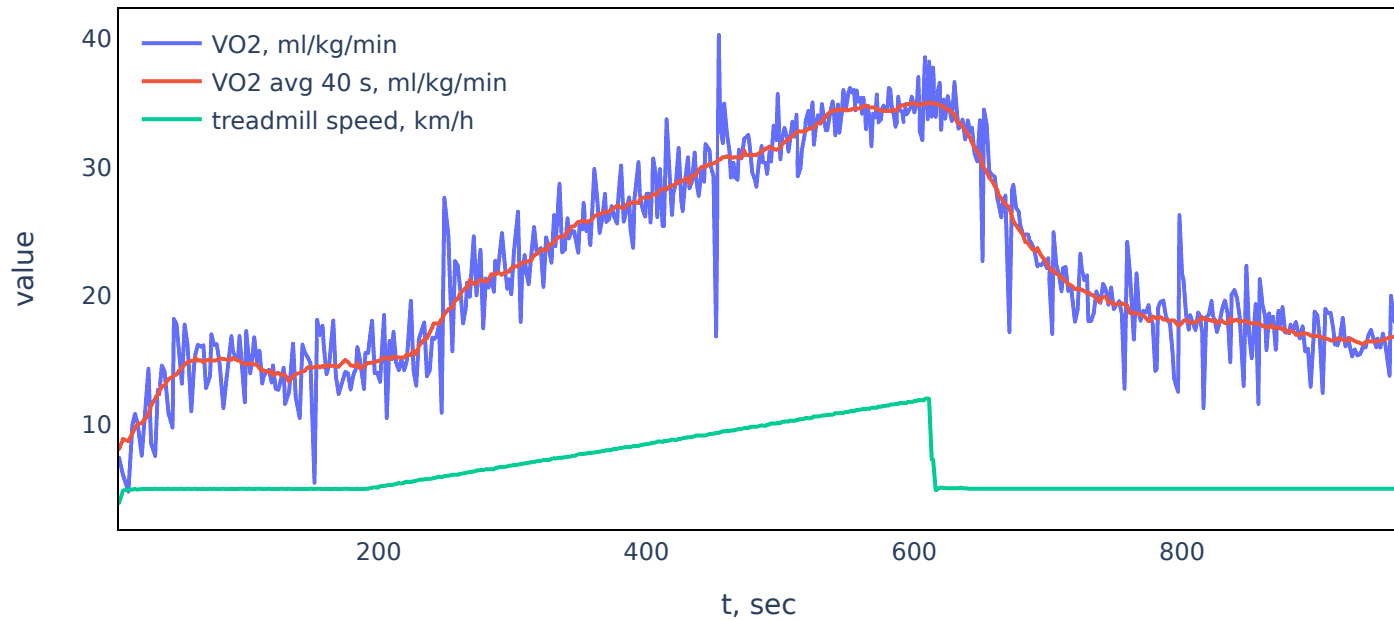

Supplement: Supplementary file 1 — Supplementary Figure S1. [file 41598_2023_43024_MOESM1_ESM.pdf]

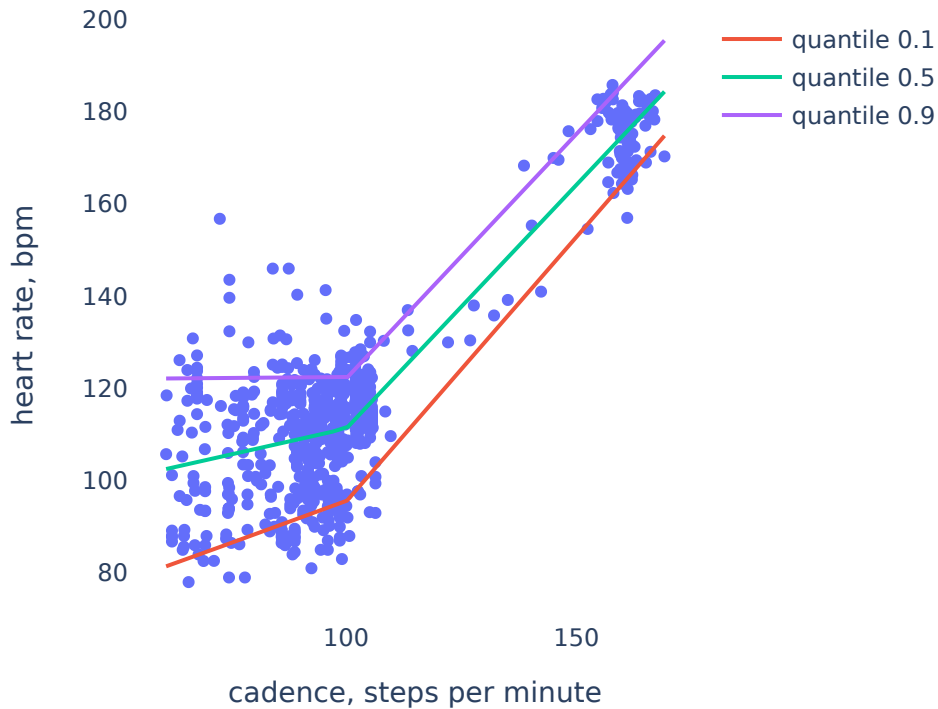

Supplement: Supplementary file 2 — Supplementary Figure S2. [file 41598_2023_43024_MOESM2_ESM.pdf]

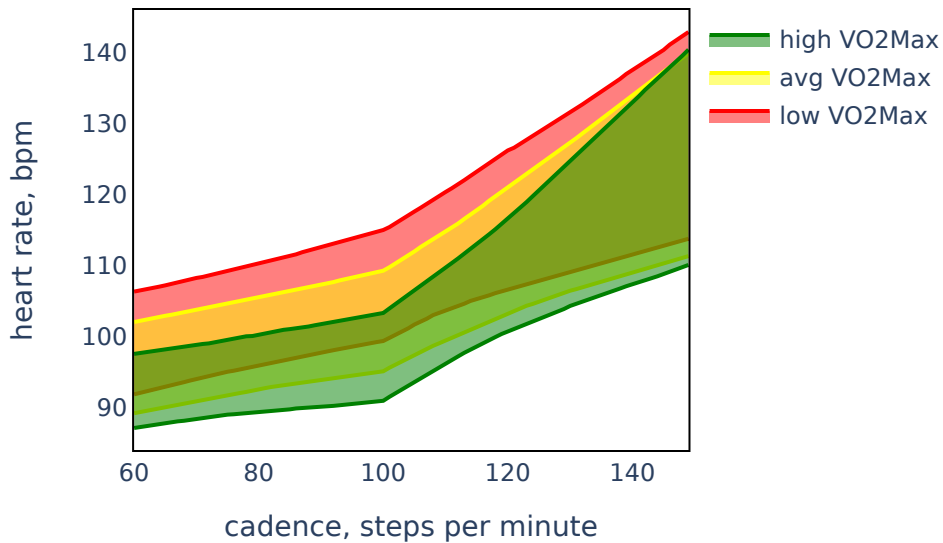

Supplement: Supplementary file 3 — Supplementary Figure S3. [file 41598_2023_43024_MOESM3_ESM.pdf]

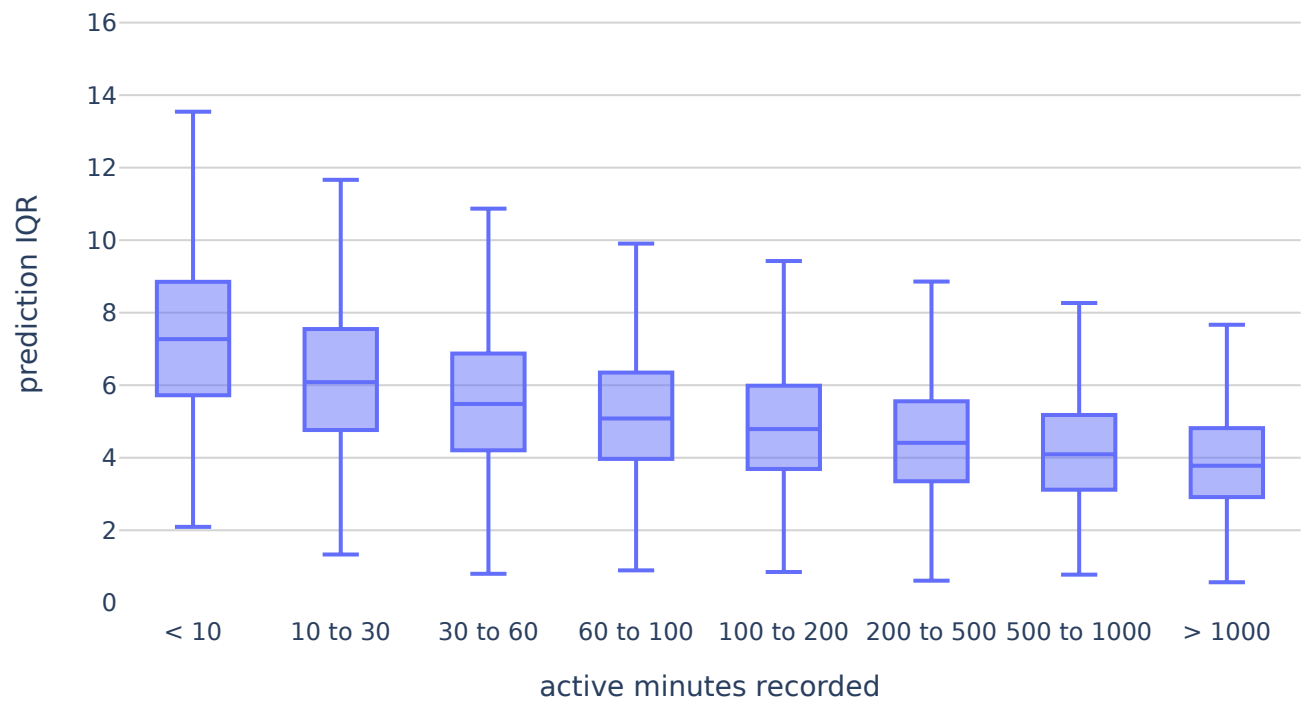

Supplement: Supplementary file 6 — Supplementary Figure S6. [file 41598_2023_43024_MOESM6_ESM.pdf]
