# Supplementary material for: Estimation of cardiorespiratory fitness using heart rate and step count data
Source: Sci Rep. 2023 Sep 22;13:15808. doi: 10.1038/s41598-023-43024-x (PMC10517160; doi:10.1038/s41598-023-43024-x)
Supplement: Supplementary file 4 — Supplementary Figure S4. [file 41598_2023_43024_MOESM4_ESM.pdf]

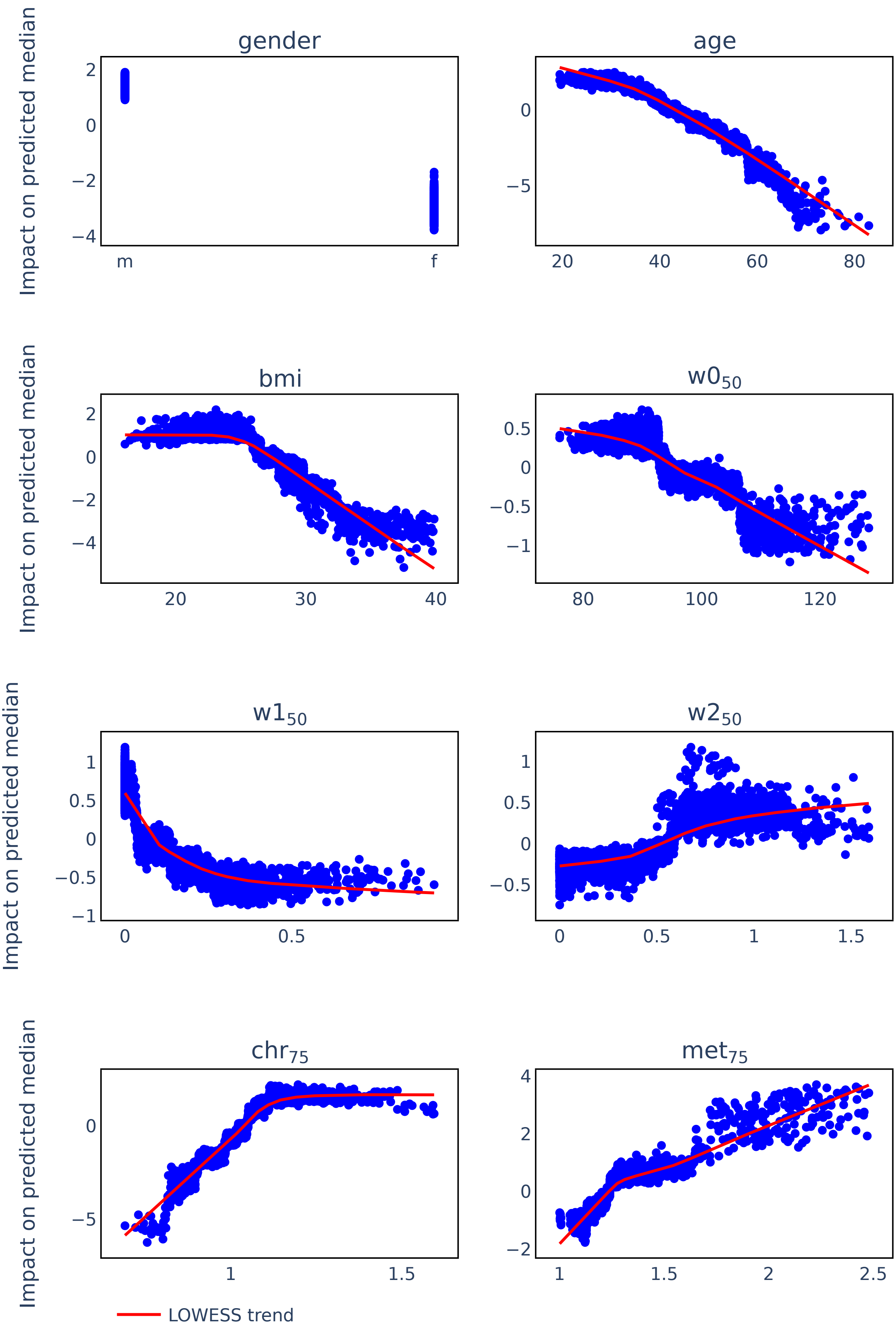
Impact on predicted median
gender
age
bmi
w0$_{50}$
w1$_{50}$
w2$_{50}$
chr$_{75}$
met$_{75}$
m
f
LOWESS trend
