# Supplementary material for: Estimation of cardiorespiratory fitness using heart rate and step count data
Source: Sci Rep. 2023 Sep 22;13:15808. doi: 10.1038/s41598-023-43024-x (PMC10517160; doi:10.1038/s41598-023-43024-x)
Supplement: Supplementary file 5 — Supplementary Figure S5. [file 41598_2023_43024_MOESM5_ESM.pdf]

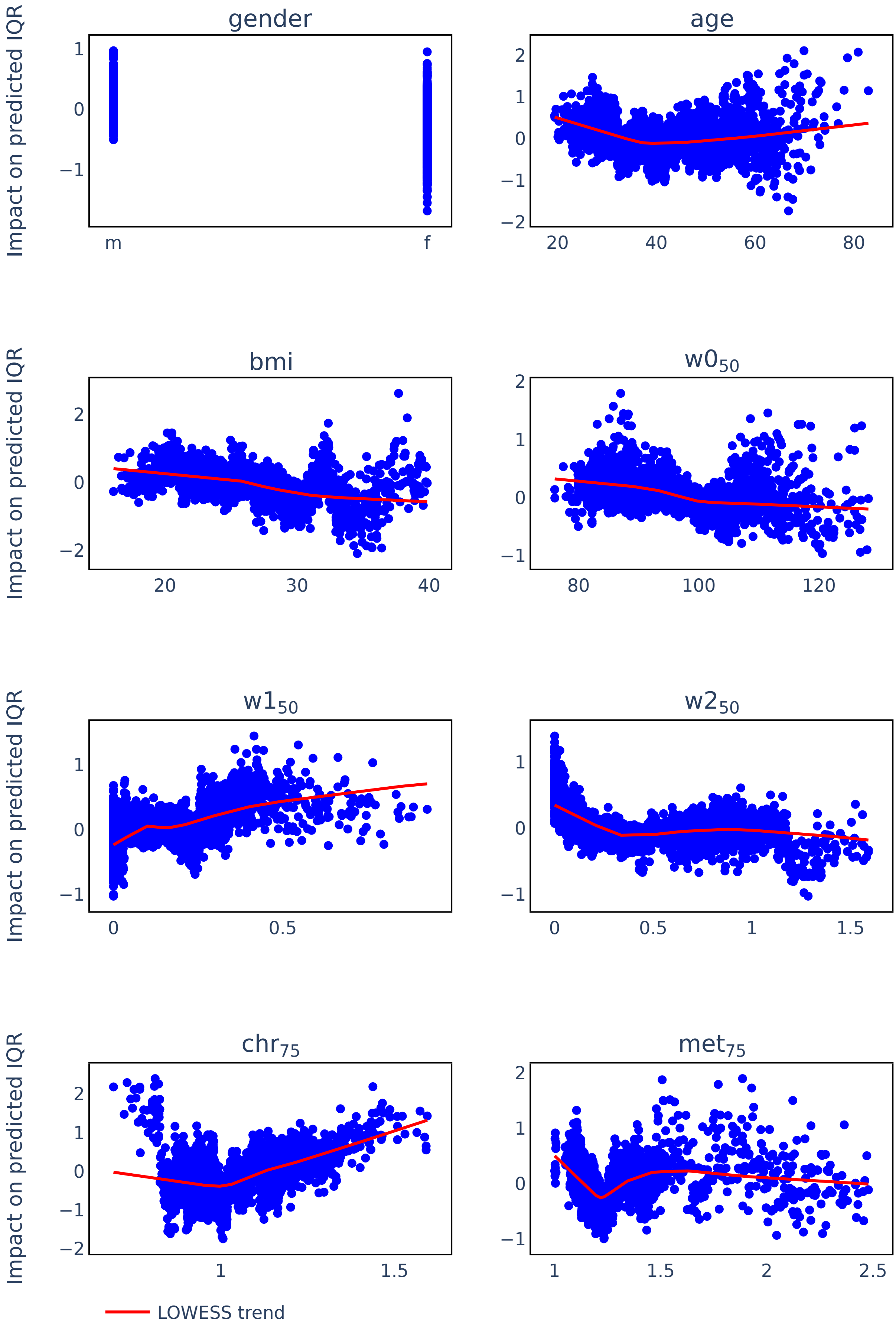
Impact on predicted IQR
gender
m
f
age
bmi
$w0_{50}$
$w1_{50}$
$w2_{50}$
chr_{75}
met_{75}
LOWESS trend
